# Tibetan medicine salidroside improves host anti-mycobacterial response by boosting inflammatory cytokine production in zebrafish

**DOI:** 10.3389/fphar.2022.936295

**Published:** 2022-08-31

**Authors:** Shumei He, Hongyan Fan, Bin Sun, Meipan Yang, Hongxu Liu, Jianwei Yang, Jianxin Liu, Sizhu Luo, Zihan Chen, Jing Zhou, Lu Xia, Shulin Zhang, Bo Yan

**Affiliations:** ^1^ Key Laboratory of Molecular Mechanistic and Interventional Research of Plateau Diseases in Tibet Autonomous Region, Key Laboratory of High Altitude Hypoxia Environment and Life Health, Joint Central Laboratory for Active Components and Pharmacological Mechanism of Tibetan Medicine, School of Medicine, Xizang Minzu University, Xianyang, China; ^2^ Shanghai Public Health Clinical Center, Fudan University, Shanghai, China; ^3^ Department of Stomatology, The First Affiliated Hospital of Shihezi University Medical College, Shihezi, China; ^4^ Medical College, China Three Gorges University, Yichang, China; ^5^ Department of Immunology and Microbiology, Shanghai Jiao Tong University School of Medicine, Shanghai, China

**Keywords:** mycobacterium, salidroside, zebrafish, innate immunity, neutrophil, macrophage, Tnfα

## Abstract

The treatment for tuberculosis (TB), especially multidrug-resistant TB (MDR-TB), has a prolonged cycle which can last up to a year. This is partially due to the lack of effective therapies. The development of novel anti-TB drugs from the perspective of host immune regulation can provide an important supplement for conventional treatment strategies. Salidroside (SAL), a bioactive component from the Tibetan medicine *Rhodiola rosea,* has been used in the treatment of TB, although its mechanism remains unclear. Here, the bacteriostatic effect of SAL *in vivo* was first demonstrated using a zebrafish–*M. marinum* infection model*.* To further investigate the underlying mechanism, we then examined the impact of SAL on immune cell recruitment during wound and infection. Increased macrophage and neutrophil infiltrations were found both in the vicinity of the wound and infection sites after SAL treatment compared with control, which might be due to the elevated chemokine expression levels after SAL treatment. SAL treatment alone was also demonstrated to improve the survival of infected zebrafish larvae, an effect that was amplified when combining SAL treatment with isoniazid or rifampicin. Interestingly, the reduced bacterial burden and improved survival rate under SAL treatment were compromised in *tnfα*-deficient embryos which suggests a requirement of Tnfα signaling on the anti-mycobacterial effects of SAL. In summary, this study provides not only the cellular and molecular mechanisms for the host anti-mycobacterial effects of the Tibetan medicine SAL but also proof of concept that combined application of SAL with traditional first-line anti-TB drugs could be a novel strategy to improve treatment efficacy.

## Introduction

TB is still the leading infectious disease cause of death by a single pathogen (exclude the impact of COVID-19), and the spread of drug-resistant TB internationally has become an urgent problem for global public health (WHO, 2021). Multidrug-resistant TB or rifampicin-resistant TB (MDR/RR-TB) usually requires extended treatment with a failure rate of 40%, and remains a serious public health concern for developing countries, such as India, Indonesia, and China (WHO, 2021). Due to the limited therapy choices and unpleasant side effects of prolonged treatment for MDR-TB and extensively drug-resistant TB (XDR-TB), the call to develop safe and effective new anti-tuberculosis drugs is compelling.

It has been well documented that the host immune response has great impact on the outcome of TB infection (Tobin et al., 2012; Roca and Ramakrishnan, 2013; Mayer-Barber et al., 2014; Boisson-Dupuis et al., 2015; Niu et al., 2022). Thus, developing novel anti-TB drugs from the perspective of immune regulation can provide an important supplement to traditional bactericidal treatment. SAL is considered to be one of the major active compound extracted from the root of *Rhodiola rosea,* a natural herbal medicine in Tibet used to alleviate high-altitude sickness (Lee et al., 2013a; Lee et al., 2013b). This root has also been used to boost the immune system (Zhao et al., 2013), as an anti-inflammatory reagent (Yang et al., 2016), to treat fatigue (Ma et al., 2015) and solid cancer (Ding et al., 2020) and as an anti-aging medication (Lu et al., 2013). The application of *Rhodiola rosea* for pulmonary infections can be traced back to the end of the eighth century AD, and it is recorded in this Medical Canon in Four Sections (Si Bu Yi Dian) (Yutuo.Yundangongbu, 1982). Studies have shown that *Rhodiola Rosea* can increase the CD4/CD8 ratio in the peripheral blood of patients with tuberculosis, which might be responsible for alleviating TB symptoms after treatment (Gang, 2009). However, whether SAL also has a protective effect during mycobacterial infection and the underlying mechanism remain unclear.

The zebrafish–*M. marinum* infection model is ideal for the study of host immune responses against mycobacterial infection (Cronan and Tobin, 2014; Benard et al., 2016; Myllymaki et al., 2016; Ramakrishnan, 2020). Zebrafish and humans have highly conserved immune systems (Trede et al., 2004; Gomes and Mostowy, 2020), and zebrafish are the natural host for *M. marinum*. About 85% virulent factors in *M. tb* can also be found in the *M. marinum* genome (Stinear et al., 2008). Particularly, zebrafish has been widely accepted to be an excellent model system for high-throughput anti-tuberculosis compound screening (Dal et al., 2021). In this study, we will take the advantages of the zebrafish–*M. marinum* infection model to explore the effect of SAL on mycobacterial infection.

## Materials and methods

### Zebrafish strains and maintenance

The wild-type (WT) AB, *Tg (mpeg1:loxP-GFP)* (Yu et al., 2017), *Tg (mpeg1:loxP-DsRedx-loxP-GFP)^hkz015t^* (Xu et al., 2016), and *tnfα* ^−/−^ (Xie Lingling et al., 2017) were maintained under standard conditions in the zebrafish facility of SPHCC as previously described (Wang et al., 2019). All embryos, except for survival experiments, were placed in egg water with 0.22 μM N-phenylthiourea (PTU) (Sigma-Aldrich) to prevent the formation of pigments (Wang et al., 2019).

### Bacterial strains and growth conditions

*M. marinum* M strain (ATCC BAA-535, labeled with Wasabi fluorescence) was grown in the 7H9 liquid medium at 32°C as previously described (Niu et al., 2019).

### Zebrafish infection

Three-dpf zebrafish embryos were infected (∼250 CFU) *via* the duct of Cuvier by micro-injection unless otherwise indicated (Niu et al., 2022).

### Drug treatment

Isoniazid (INH) (Aladdin), rifampicin (RIF) (Sigma), and SAL (Shidande) were dissolved in egg water to make working concentrations. For the early myeloid cell development and wound response experiments, the embryos were treated with SAL 36 h before the assays. For infection with *M. marinum*, the embryos were treated with INH, RIF, or SAL after bacterial injection.

### Acute zebrafish toxicity test

For this test, 3-dpf wild-type embryos were treated with different concentrations of INH and SAL. Feeding started at 5 dpf with a dry larval diet (AP100, Zeigler) twice per day, and the development or death of the embryos was monitored daily until 8 dpf.

### Minimum inhibitory concentration determination

MIC experiments were performed using a 7H10 agar plate (BD) with a serial of SAL and INH concentrations. The plates were grown at 32°C for about 8 days and then observed for colony growth (Wiegand et al., 2008).

### Cell culture and infection

Raw264.7 cells were cultured in 100 mm cell culture dishes, and SAL (1.6 mM) was added 36 h before infection. The Raw264.7 cells were plated in 24-well plates at 3 × 10^5^ cells/well 12 h before infection and then infected with a single-cell suspension of green fluorescent-labeled *M. marinum*-Wasabi (MOI = 1). The infected Raw264.7 cells were incubated at 32°C and 5% CO_2_. The plates were imaged using BioTek Cytation 5. Images were taken with a ×20 objective at 2 hpi, 24 hpi, and 48 hpi.

### Tail amputation assay

The embryos were pretreated with 1.6 mM SAL or vehicle at 1.5 dpf. The embryos were placed in egg water with 0.02% tricaine (Sigma-Aldrich) in a sterile Petri dish, and tail fin amputation was performed using a sterile 24-gauge needle (Yan et al., 2014). RNA was extracted from the caudal fin for further analysis of cytokine expression.

### Infection-induced macrophage and neutrophil recruitment assay

For infection-induced macrophage and neutrophil recruitment assay, embryos were pre-treated with 1.6 mM SAL or vehicle 36 h before the assays. Three-dpf zebrafish embryos were anesthetized with tricaine (Sigma, 200 μg/ml) and mounted in 1% agarose for subcutaneous infection (∼300 CFU) (Niu et al., 2019). After infection, the embryos were washed into pre-warmed egg water and placed into a 28.5°C incubator immediately. The embryos were fixed at 2 hpi and then applied for macrophage and neutrophil staining assays.

### Sudan black staining

The embryos were fixed for 2 h at room temperature and then washed with PBST for 4 × 5 min. The embryos were then stained with Sudan Black (Sangon Biotech) staining solution for 25 min at room temperature and washed with 70% ethanol as previously reported (Wang et al., 2019).

### Antibody staining

The antibody staining was performed as previously described (Wang et al., 2019). Briefly, the 3-dpf embryos were fixed and then stained with the goat polyclonal anti-GFP (Abcam, AB6658, 1:500) or anti-DsRed (TaKaRa, 632496, 1:500) primary antibody. The primary antibody was washed out and then stained with AF488 donkey anti-goat (Invitrogen, A11055, 1:500) or AF594 donkey anti-rabbit IgG (H + L) (YEASEN, A05121, 1:500) secondary antibodies, accordingly.

### Imaging and image analysis

Fluorescent images were taken by Leica DMi8 microscopy. Bacterial burden was quantified by measuring the integrated fluorescent intensity of Wasabi-labeled bacteria with Fiji ImageJ software. Fluorescent-labeled macrophage areas were calculated by measuring the fluorescent area with Fiji ImageJ software.

### Survival curve

Embryos were infected with *M. marinum*: Wasabi (∼250 CFU) and placed in egg water with different concentrations of SAL, INH, RIF, SAL plus INH, and SAL plus RIF. Feeding started at 5 dpf with the AP100 larval diet (Zeigler) twice per day. The numbers of dead zebrafish were recorded every day until 14 dpi.

### Quantitative RT-PCR

Total RNA was extracted from zebrafish wounded caudal fins or whole embryos using TRIzol (Ambion). The PrimeScript reverse transcription kit (TaKaRa) was used for cDNA synthesis, and quantitative RT-PCR was performed using SYBR Green Supermix (Promega) to determine relative gene expression. The primer sequences (Genewiz) used are as follows: *ef1a*-F: 5′-CTT​CTC​AGG​CTG​ACT​GTG​C-3′; *ef1a*-R: 5′-CCG​CTA​GCA​TTA​CCC​TCC-3′; *il1β*-F: 5′GTA​CTC​AAG​GAG​ATC​AGC​GG-3′; *il1β*-R: 5′-CTC​GGT​GTC​TTT​CCT​GTC​CA-3′; *tnfα*-F: 5′-GCT​GGT​GAT​AGT​GTC​CAG​GAG-3′; *tnfα*-R: 5′-TTG​ATT​GCC​CTG​GGT​CTT​ATG-3′; *il6*-F: 5′-CCT​CAA​ACC​TTC​AGA​CCG​CT-3′; *il6*-R: 5′-CTG​GCT​GTT​TAT​GGC​CTC​CA-3′; *ccl2*-F: 5′-GTC​TGG​TGC​TCT​TCG​CTT​TC-3′; *ccl2*-R: 5′- TGC​AGA​GAA​GAT​GCG​TCG​TA-3′; *cxcl8-l1*-F: 5′-TGT​TTT​CCT​GGC​ATT​TCT​GAC​C-3′; *cxcl8-l1*-R: 5′-TTT​ACA​GTG​TGG​GCT​TGG​AGG​G-3′; *cxcl8-l2*-F:5′-CCACACACACTCCACACACA-3′; *cxcl8-l2*-R: 5′- CCA​CTG​AAT​TGT​CCT​TTC​ATC​A-3′; *cxcl11*a-F: 5′-ACT​CAA​CAT​GGT​GAA​GCC​AGT​GCT-3′; and *cxcl11*a-R: 5′-CTT​CAG​CGT​GGC​TAT​GAC​TTC​CAT-3′ (Yan et al., 2014; Wang et al., 2019; Sommer et al., 2021).

### Statistical analysis

GraphPad Prism 8.0.2 was applied for statistical analyses. The results are shown as mean ± SEM. Differences were analyzed by using the two-tailed student’s t-test for comparisons between two groups, and a one-way/two-way ANOVA for multiple groups. Zebrafish mortality and survival data were analyzed using the log rank (Mantel–Cox) test (Dong et al., 2021). Statistical significance was indicated as follows: *, *p* < 0.05; **, *p* < 0.01; ***, *p* < 0.001; and ns, not statistically significant (*p* > 0.05).

## Results

### Salidroside treatment does not cause gross developmental defects in zebrafish embryos

**FIGURE 1:**
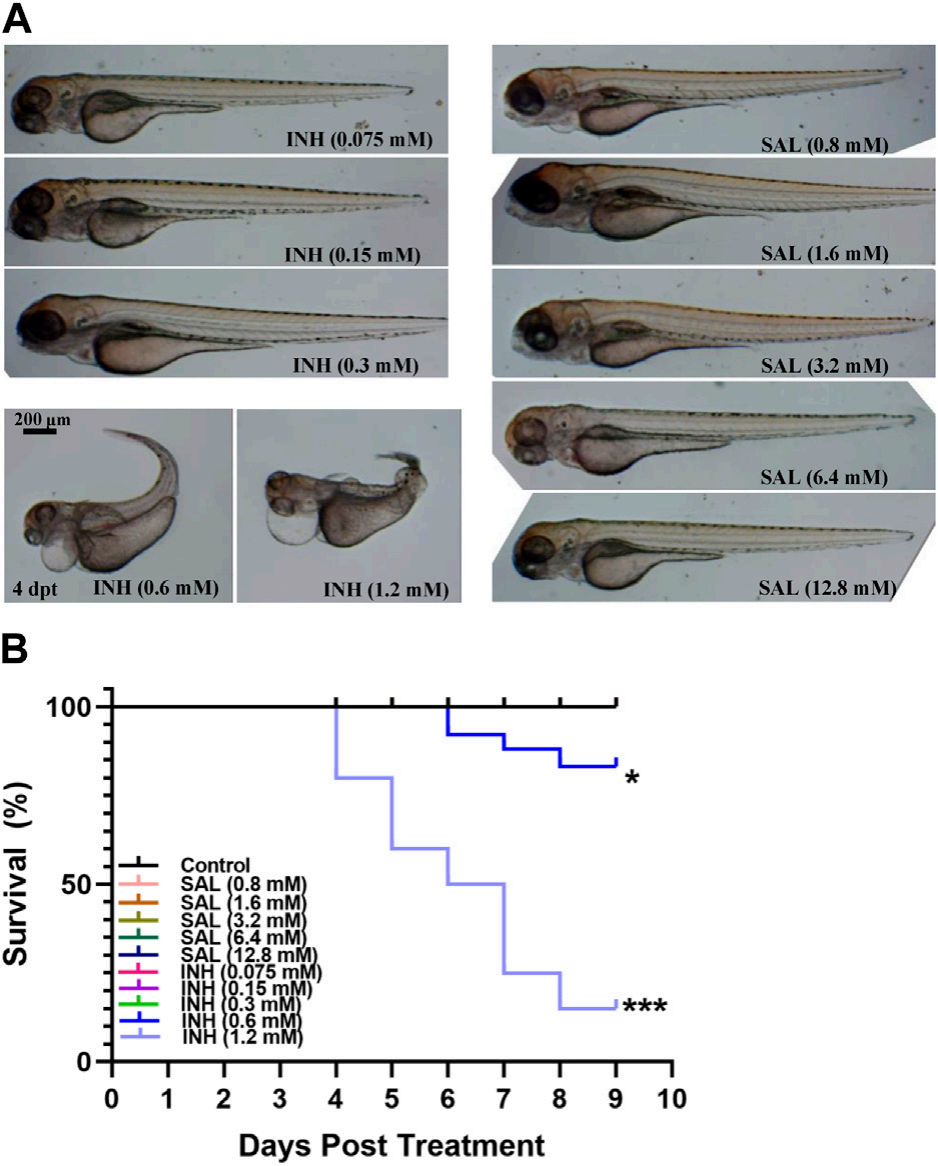
SAL has no obvious teratogenic effect on the growth and development of zebrafish. **(A)** Representative photos for zebrafish embryos exposed to different concentrations of INH and SAL (4 dpt). **(B)** Mortality rates of zebrafish embryos exposed to different concentrations of INH and SAL from 0 to 8 dpf. A and B; *n* = 20 per group. This is one representative data from three independent repeats.

Because toxic side effects often appear after prolonged administration of anti-TB drugs (Nunn et al., 2019), the toxicity of SAL on zebrafish embryos was first assessed using INH as the control. Based on previous reports and our preliminary results (Dharra et al., 2017; Reingewertz et al., 2020), we first selected the ranges of 0.075–1.2 mM and 0.8–12.8 mM for toxicity testing, respectively. A dose-dependent toxicity effect can be observed with concentrations of INH above 0.6 mM, while treatment with SAL showed no death or deformity phenotypes (Figures 1A,B; Supplementary Table S1). These results indicate that SAL treatment (≤12.8 mM) has no toxic effects on zebrafish embryonic development.

### Salidroside inhibits *M. marinum* proliferation *in vivo*

**FIGURE 2:**
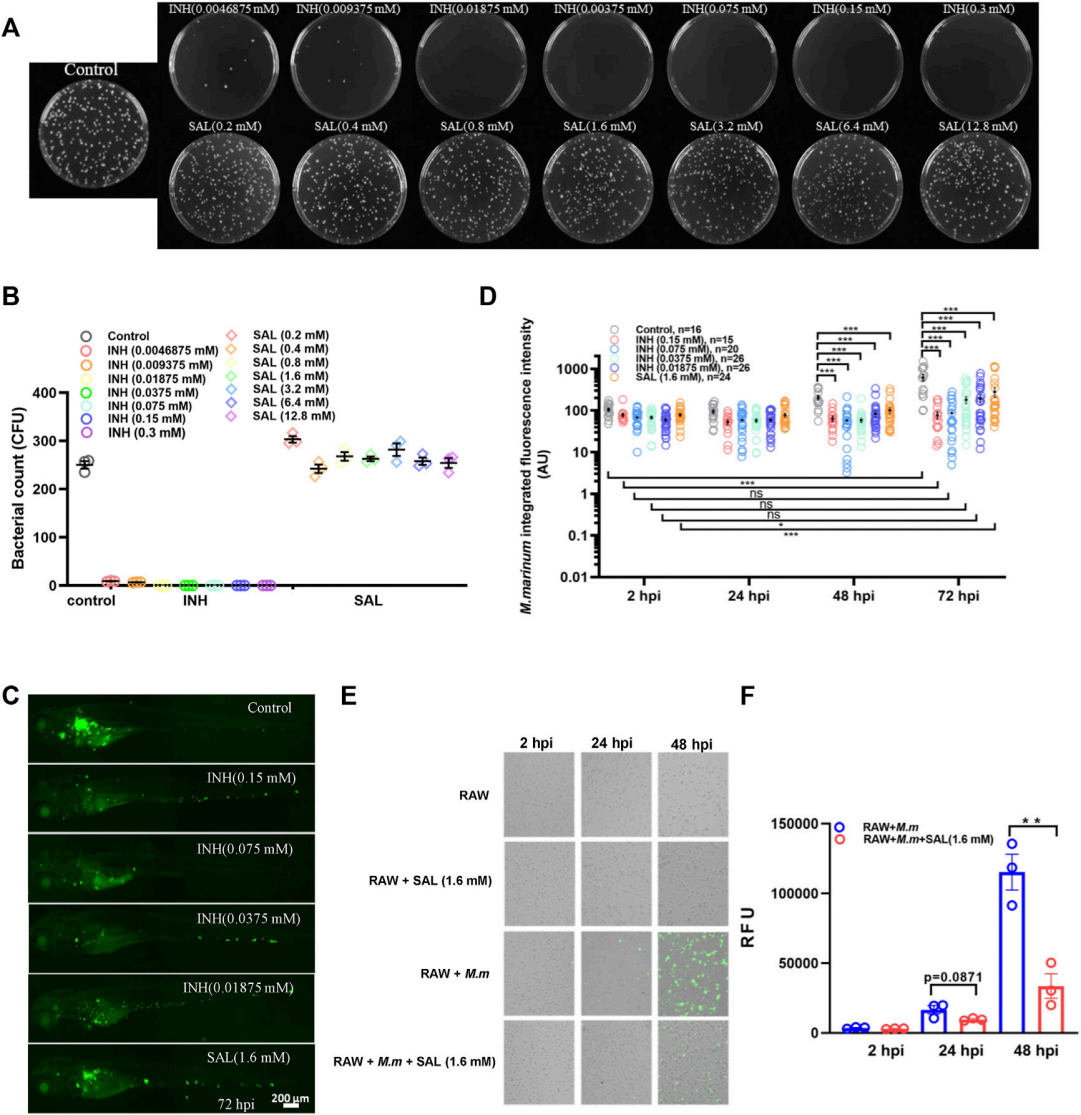
SAL has bacteriostatic activity *in vivo* but not *in vitro*. **(A)** Images showing the growth of *M. marinum* on 7H10 plates containing different concentrations of INH and SAL after 8 days incubation. **(B)** Statistics of the colony number in **(A)**. **(C)** Representative images for the distribution of *M. marinum* (Wasabi) in different groups of zebrafish at 72 hpi. Scale bar: 200 μm. **(D)** Statistics of integrated green fluorescence intensity in **(C)**. Values are shown as mean ± s.e.m. One-way ANOVA for comparison at the same time point. Two-way ANOVA for co-operation at different time points. **(E)** *M. marinum-*Wasabi-infected RAW cells with or without SAL treatment. **(F)** Statistics of relative fluorescence units (RFU) of total (extracellular and intracellular) bacteria in **(E)**. A and B are one representative data from three independent repeats. C and D are one representative data from two independent repeats. E and F are one representative data from three independent repeats.

In order to study the impact of SAL on mycobacterial infection, we first tested whether SAL has direct anti-mycobacterial activity *in vitro*. *M. marinum* was cultured on 7H10 agar plates containing different concentrations of INH and SAL for about 8 days to determine the MIC (Figures 2A,B). The results showed the INH MIC to be 0.01875 mM, similar to that reported previously (Reingewertz et al., 2020). However, SAL showed no direct anti-mycobacterial activity regardless of concentration. The *in vivo* anti-mycobacterial activity of SAL was next assessed using a zebrafish–*M. marinum* infection model. INH (≥0.0375 mM) showed significant dose-dependent bactericidal activity. However, SAL treatment only showed a slight but significant decrease in green fluorescence intensity (Figures 2C,D; Supplementary Figure S1). The anti-mycobacterium effect of SAL was also confirmed by a rodent macrophage infection model (Figures 2E,F). Together, these data suggest SAL may convey bacteriostatic activity *via* the host immune system.

### Salidroside treatment promotes recruitment of macrophages and neutrophils to the wound and infection sites

**FIGURE 3:**
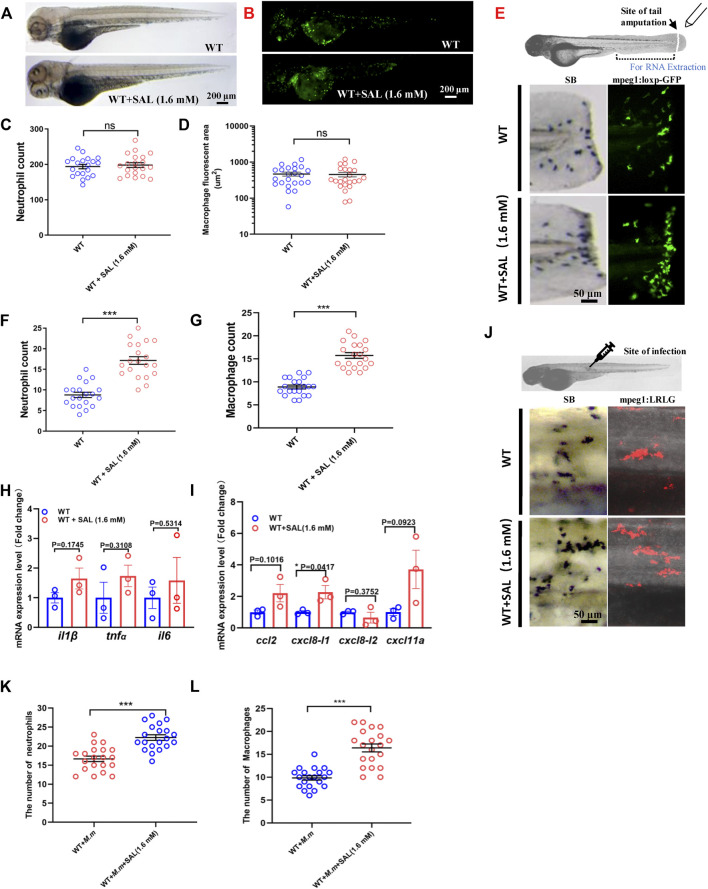
SAL promotes the recruitment of innate immune cells to the wound and infection sites. **(A)** Images of whole body SB staining of 3-dpf embryos. **(B)** Fluorescent images of 3-dpf *Tg (mpeg1:loxP-GFP)* embryos. A and B Scale bar: 200 μm. **(C)** Statistics of the number of SB^+^ neutrophils in A. **(D)** Quantification of total macrophage fluorescent area in B. **(E)** Schematic view of tail amputation experiment and the tissue for RNA extraction (upper panel); the recruitment of SB^+^ neutrophils to wound (left panel); and the recruitment of mpeg1:loxp:GFP^+^ macrophages to wound (right panel). Scale bar: 50 μm. **(F)** Statistics of the number of SB^+^ neutrophils in E. **(G)** Statistics of the number of macrophages in E. **(H)** Expression of various cytokines in the tail region at 2 hpa. **(I)** Expression of macrophage and neutrophil recruitment-related chemokines in the tail region at 2 hpa. **(J)** Images of the SB^+^ neutrophils (left panel) and mpeg1:LRLG^+^ macrophages (right panel) infiltration to infection sites. Scale bar: 50 μm. **(K)** Statistics of the number of SB^+^ neutrophils in J. **(L)** Statistics of the number of macrophages in J. A–G, J–L: *n* = 20 per group; H and I: *n* = 30 per sample. This is one representative data from three independent biological replicates.

To confirm our hypothesis that SAL inhibits bacterial proliferation *via* host immune regulation, we further investigated the impact of SAL on immune system development and wound response. No significant difference was observed in neutrophil and macrophage numbers between SAL-treated and control groups (Figures 3A–D), which suggests that SAL treatment does not have a major effect on early myeloid development. We then looked into the effects of SAL on the immune response during non-infectious injury. Interestingly, the numbers of neutrophils and macrophages recruited to wound sites in the SAL treatment group were significantly higher than those in the untreated group (Figures 3E–G). We further found that the expression of pro-inflammatory cytokines and chemokines was increased in the SAL-treated group (Figures 3H,I). Consistently, an increased macrophage and neutrophil infiltration was also observed after SAL treatment (Figure 3J–L). Our data suggest that SAL does not impact general myeloid development but can promote innate immune cell recruitment in response to injury and infection.

### The combined application of salidroside and first-line anti-TB drugs significantly improves survival in *M. marinum*-infected zebrafish

**FIGURE 4:**
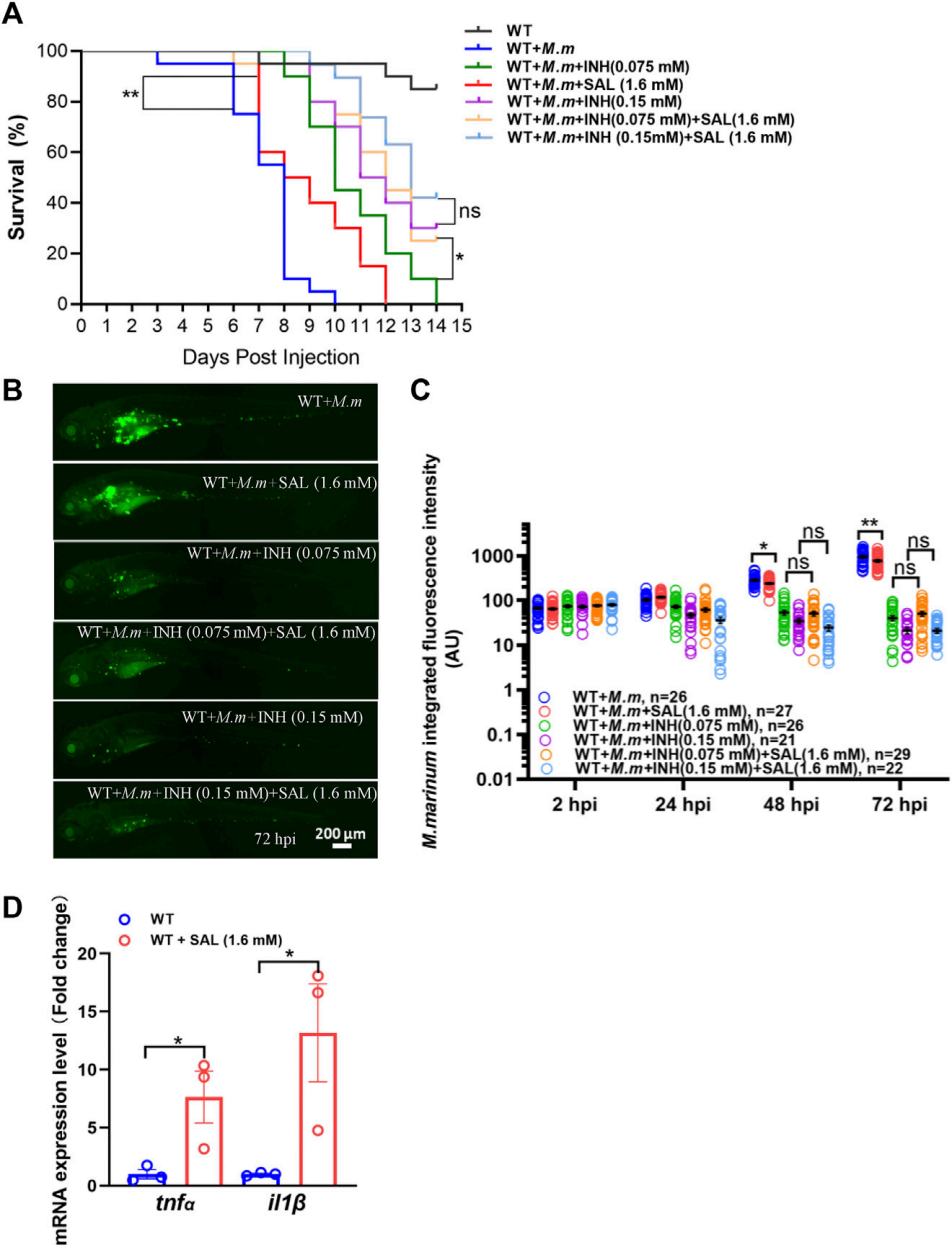
Combined treatment of SAL and INH significantly improve the survival of zebrafish during mycobacterial infection. **(A)** Survival rates of *M. marinum*-infected zebrafish embryos treated with various concentrations of INH and SAL. *n* = 20 per group. **(B)** Representative images for the distribution of *M. marinum* (Wasabi) in different groups of zebrafish embryos at 72 hpi. Scale bar: 200 μm. **(C)** Statistics of integrated green fluorescence intensity in **(B)**. One-way ANOVA was applied for the comparison. **(D)** Expression of two potent pro-inflammatory cytokines at 12 hpi. *n* = 30 per sample. A and D: data are representative of three independent biological replicates. B and C data are representative of two independent repeats.

Due to the different anti-mycobacterial mechanisms of each drug, we speculate that the combination of SAL and first-line anti-TB drugs may improve anti-mycobacterial efficacy. Bacterial burdens and survival curves were monitored after single or combined treatments. Consistent with the *in vivo* bacteriostatic activity, SAL treatment could only significantly improve the survival rates (Figure 4A). However, the bacterial fluorescence intensity did not show a significant difference between INH only and combined treatment groups (Figures 4B,C). Excitingly, although the combination of INH and SAL improved the survival rates as compared with INH treatment alone, regardless of the INH concentration, this improvement was only significant in low-concentration INH-treated groups (Figure 4A). A similar effect on survival rates was also observed when zebrafish embryos were treated with RIF and SAL (Supplementary Figure S2). These results imply that the improvement in bacterial clearance and increase in survival by the combined application of first-line anti-TB therapies with the traditional Tibetan medicine SAL may be due to their distinct anti-mycobacterial mechanisms.

### Salidroside promotes the production of pro-inflammatory cytokines during infection

The success of the host anti-tuberculosis response depends on tight control of the expression of pro-inflammatory cytokines such as TNFα and IL1β (Cooper et al., 2011). Thus, we examined RNA expression of these potent cytokines in infected zebrafish embryos after SAL treatment and observed a significant increase in *tnfα* and *il1β* compared to controls (Figure 4D). These findings suggest that the potential mechanism of SAL treatment against mycobacterial infection is inducing upregulation of pro-inflammatory cytokines.

### Salidroside-mediated host protective effect during mycobacterial infection requires Tnfα signaling

**FIGURE 5:**
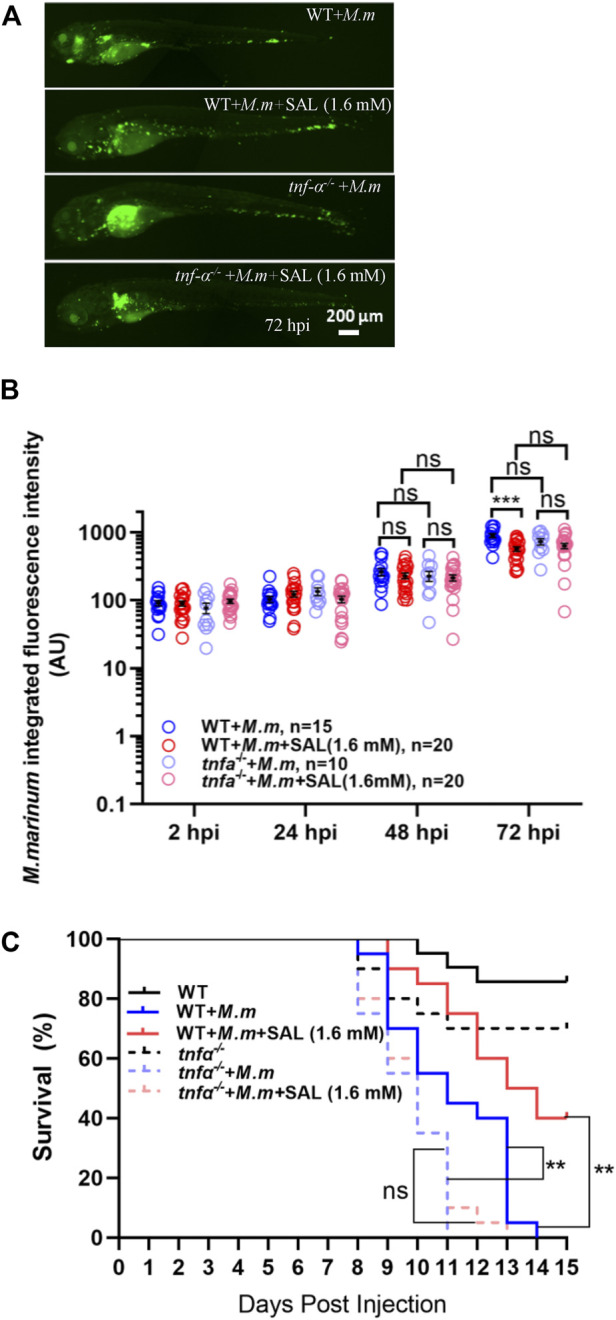
Tnfα is required for SAL-mediated protective effects during mycobacterial infection. **(A)** Representative images for the distribution of *M. marinum* (Wasabi) in different groups of zebrafish embryos at 72 hpi. Scale bar: 200 μm. **(B)** Statistics of normalized green fluorescence intensity in A. One-way ANOVA was applied for the comparison. **(C)** Survival rates of *M. marinum*-infected zebrafish *tnfα* ^−/−^ embryos treated with various concentrations of INH and SAL. *n* = 20 per group. A, B, and C: data are representative of three independent repeats.

Because Tnfα is a potent pro-inflammatory cytokine associated with mycobacterial infection, we tested whether the SAL-mediated protective effect required Tnfα signaling. Interestingly, both the differences between treatments in bacterial fluorescence intensity and the survival rates were diminished in *tnfα*-deficient embryos (Figures 5A–C), indicating that Tnfα signaling is required for SAL-mediated protection during mycobacterium infection.

## Discussion

TB is one of the major infectious diseases endangering public health. Given the long treatment cycle and continual outbreaks of drug-resistant TB, there is an urgent need for the development of novel anti-TB therapies. SAL is a prominent bio-active extract of *Rhodiola rosea* and has been reported to play an important role in treating many immune system-related diseases (Qiu and Jia, 2014; Zheng et al., 2015; Hao et al., 2019). However, research concerning the application of SAL for the treatment of TB is limited. In this study, we used a zebrafish–*M. marinum* infection model to study the impact of SAL on mycobacterial infection. Our data show that while SAL has no direct anti-mycobacterial activity *in vitro*, it can significantly inhibit the bacterial proliferation *in vivo* and increase the survival of *M. marinum*-infected zebrafish. Excitingly, the combination of SAL and INH achieved better protective effects in controlling bacterial burden and improving the survival curve than either treatment alone. We have also shown that SAL can regulate immune cell recruitment during wound response and infection. The increased expression of pro-inflammatory cytokines and innate immune cell infiltration during infection could be the mechanisms of action for SAL’s protective effects during mycobacterial infection *in vivo*.

SAL has been reported to play a role in boosting the immune system. For example, it has been reported that adding SAL to the diet can enhance resistance against *Aeromonas hydrophila* infection in crucian carp (*Carassius auratus*) (Yang et al., 2020). Similarly, Navita Sharma et al. (2016) reported that SAL exerts its antiviral activity by increasing the expression of RNA helicases such as RIG-I, thereby initiating a downstream signaling cascade that induces upregulation of IRF-3 and IRF-7. However, the underlying molecular mechanisms for the SAL’s anti-infection role remain unclear. In this study, we first found that SAL is able to promote the recruitment of neutrophils and macrophages to the wound and infection sites and then demonstrated a positive regulatory role of SAL for the expression of pro-inflammatory cytokines during mycobacterial infection. Considering that zebrafish embryos only preserve the innate immune system, we can only dissect the impact of SAL on the innate immune system. Whether SAL also has a regulatory effect on adaptive immunity is worth further exploration.

In this study, we have observed significantly increased expression of pro-inflammatory cytokines after SAL treatment during infection, including *tnfα* and *il1β*. However, we did not observe a similar increase pattern during a wound response. It is possible that, compared with the impact of SAL on mycobacterial infection, which is a systematic response, the induced cytokine response to a localized tail amputation is too trivial to be detected. Similar findings were observed while studying the disease resistance role of SAL in common carp (*Cyprinus carpio*) and rainbow trout (*Oncorhynchus mykiss*) (Baba et al., 2018; Zemheri-Navruz et al., 2019). However, the research results of Guan et al. (2011) showed that SAL significantly reduces the production of TNFα, IL1β, and IL6 in the serum of mice stimulated by LPS. This inconsistency may be due to the different animal models chosen and the inflammatory stimuli applied. Thus, whether SAL plays distinct roles in different disease models requires further verification.

By utilizing *tnfα*-deficient zebrafish, we proved that the protective effect of SAL during *M. marinum* infection requires *tnfα* signaling (Figure 5C). We still cannot exclude the possibility that *il1β*, another significantly increased mycobacterial infection-related cytokine*,* also plays a role in SAL-mediated protection, which is worth further exploration in the future. Interestingly, there is no significant difference in the bacterial burden during the early stage of infection between *tnfα*-deficient embryos and wild-type controls, which is also consistent with previous reports (Figure 5B) (Clay et al., 2008; Xie Lingling et al., 2017). It is probably due to the dual role of *tnfα* on host control of infection and tissue damage (Tobin et al., 2010; Roca and Ramakrishnan, 2013). In an osteoarthritis rat model, it was found that SAL can regulate the inflammatory and immune responses of the rats through the NF-κB pathway (Gao et al., 2020). This suggests that SAL may act on host anti-mycobacterial responses *via* the NF-κB–Tnfα axis. Interestingly, the impact of SAL on the anti-mycobacterial responses does not work in a simple dose-dependent manner. No significant improvement in the anti-mycobacterial effect was further observed upon increasing the dose, once the concentration of SAL reaches the effective concentration. It suggests that a more complicated regulatory mechanism may exist. A balance in TNFα signaling has also been suggested as a requirement for successful control of mycobacterial proliferation and granuloma formation both in zebrafish and mouse models (Roach et al., 2002; Tobin et al., 2010; Dorhoi and Kaufmann, 2014). Whether SAL has an impact on granuloma formation also deserves further study.

The most widely used first-line therapy for TB is a combination of isoniazid, rifampin, pyrazinamide, and ethambutol, which mainly focuses on bacterial control. However, this treatment cycle is long and often results in treatment-induced drug resistance and severe side effects. Application of traditional Chinese medicine for TB shifts the focus on the immune regulation of the host (Choi et al., 2015). Thus, the combined application of traditional Chinese medicine and conventional anti-TB drugs may have the advantage of a shorter treatment cycle and avoiding therapy-induced drug resistance. In this study, we have applied fluorescent-labeled *M. marinum* to carry out serial observations of bacterial burdens both in zebrafish and RAW264.7 macrophage infection models. Compared with conventional plating and counting for the CFU method, fluorescence-based methods can greatly reduce the time and experimental resource costs (Takaki et al., 2012; Cortesia et al., 2017; Hodges et al., 2018). Excitingly, the combined usage of SAL and INH showed the most promising therapeutic results in this study. Interestingly, we did not observe a reduction of bacterial burden in the SAL and INH combined treatment group compared with the INH only group, which may be due to the time point we chose for detecting bacterial burden. A reduced bacterial burden in the combined treatment group might be detected at a later stage of infection. Notably, we have also explored the protective effect of the combined application of SAL and RIF in the revised article and found a similar effect to that produced by the treatment of SAL and INH. In summary, our results provide a proof of concept that the combination of conventional anti-TB and traditional Chinese medicine is a superior treatment strategy.

In summary, SAL was proven to play an anti-infection role in a zebrafish–*M. marinum* infection model. We have also shown that SAL treatment can regulate innate immune cell recruitment and upregulate the expression of pro-inflammatory genes. We further demonstrated, by using a *tnfα*-deficient mutant zebrafish model, that the protective role of SAL requires Tnfα signaling. Our data provide proof of concept that immune-regulating traditional Tibetan medicine could be a potent tool in anti-TB drug development.

## Data Availability

The original contributions presented in the study are included in the article/Supplementary Material; further inquiries can be directed to the corresponding authors.
