# Supplementary figures and images for: Tibetan medicine salidroside improves host anti-mycobacterial response by boosting inflammatory cytokine production in zebrafish
Source: Front Pharmacol. 2022 Aug 31;13:936295. doi: 10.3389/fphar.2022.936295 (PMC9470765; doi:10.3389/fphar.2022.936295)

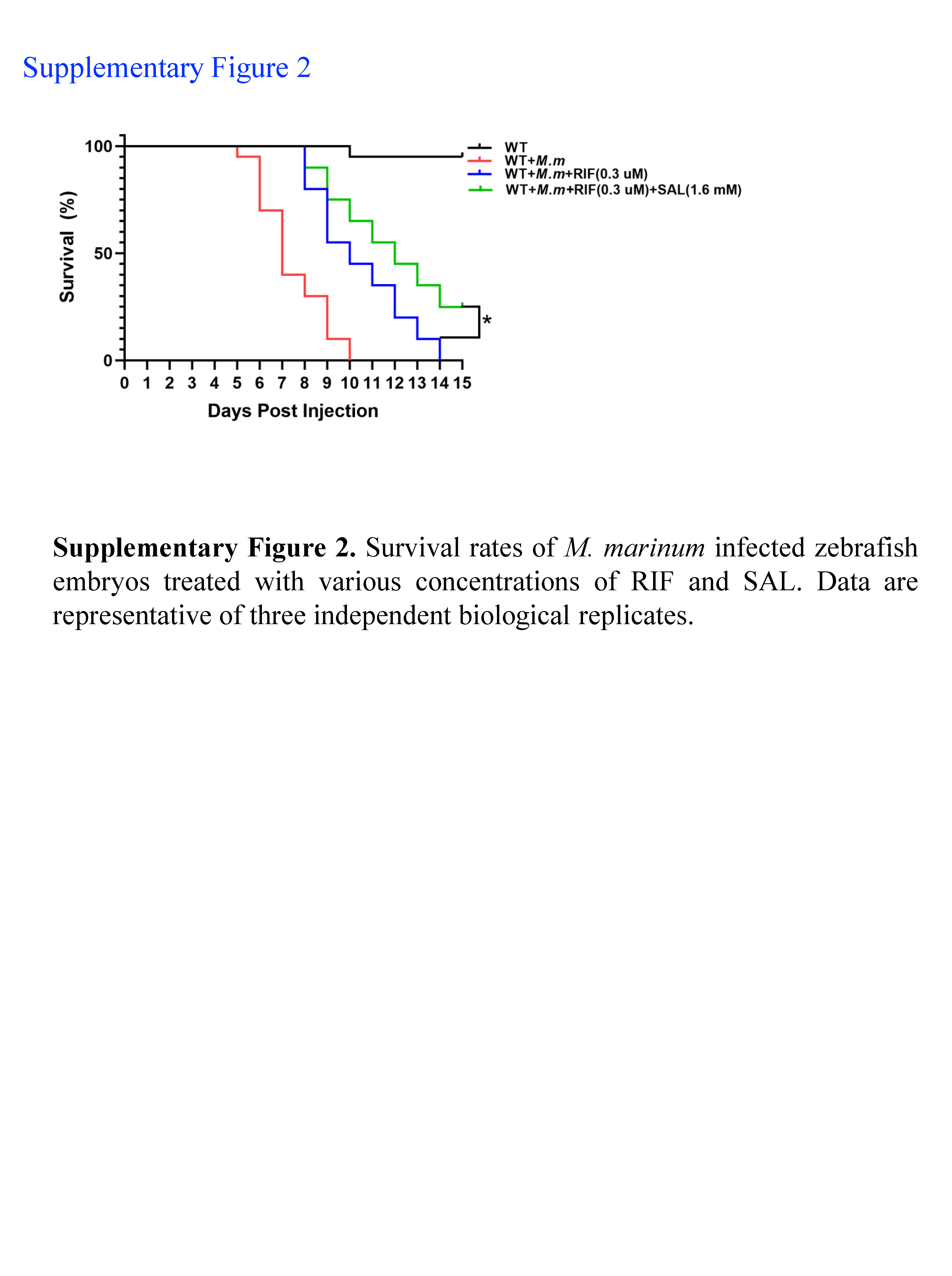

Supplement: Supplementary file 1 [file Image3.TIFF]

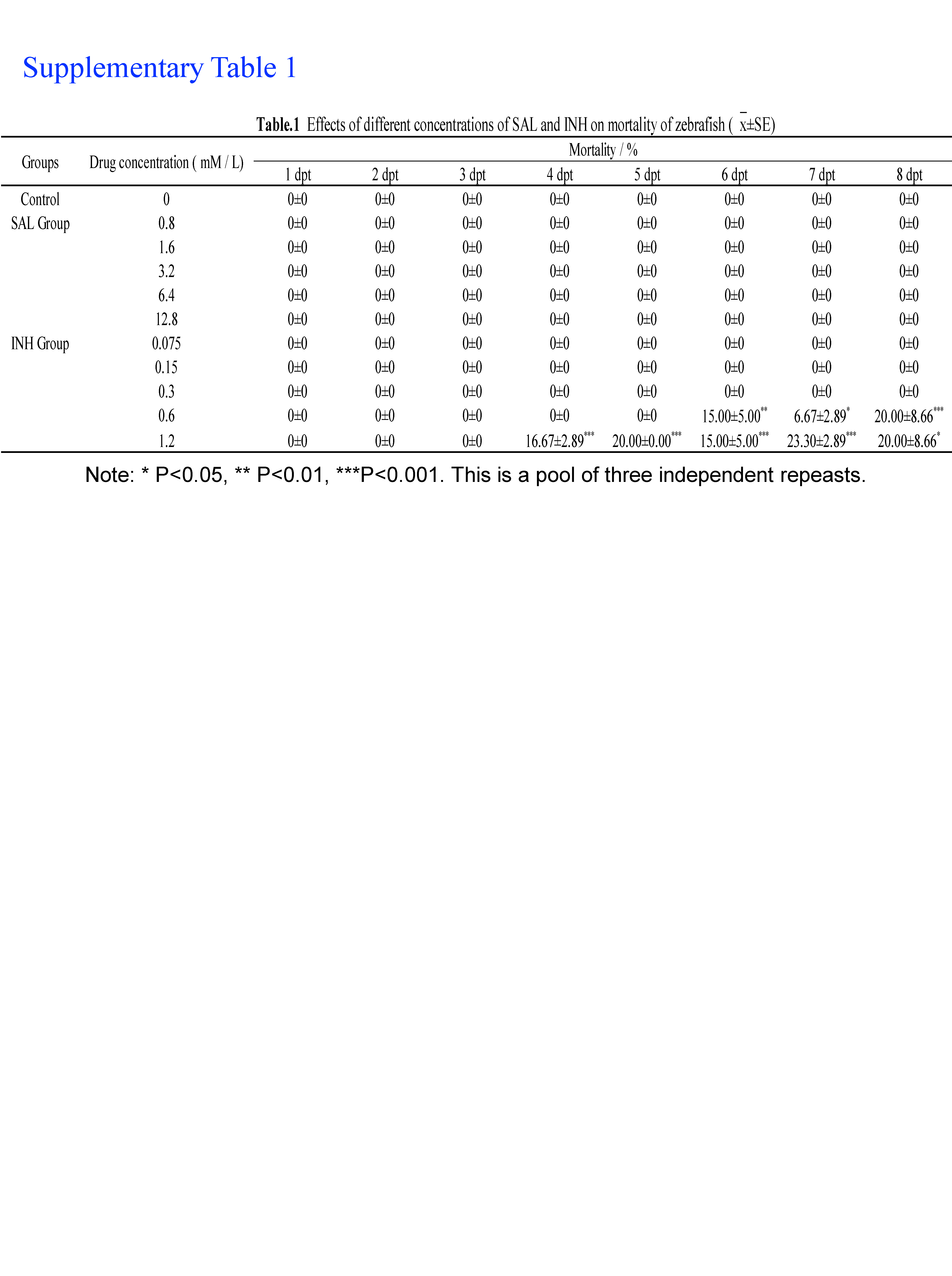

Supplement: Supplementary file 2 [file Image1.TIFF]

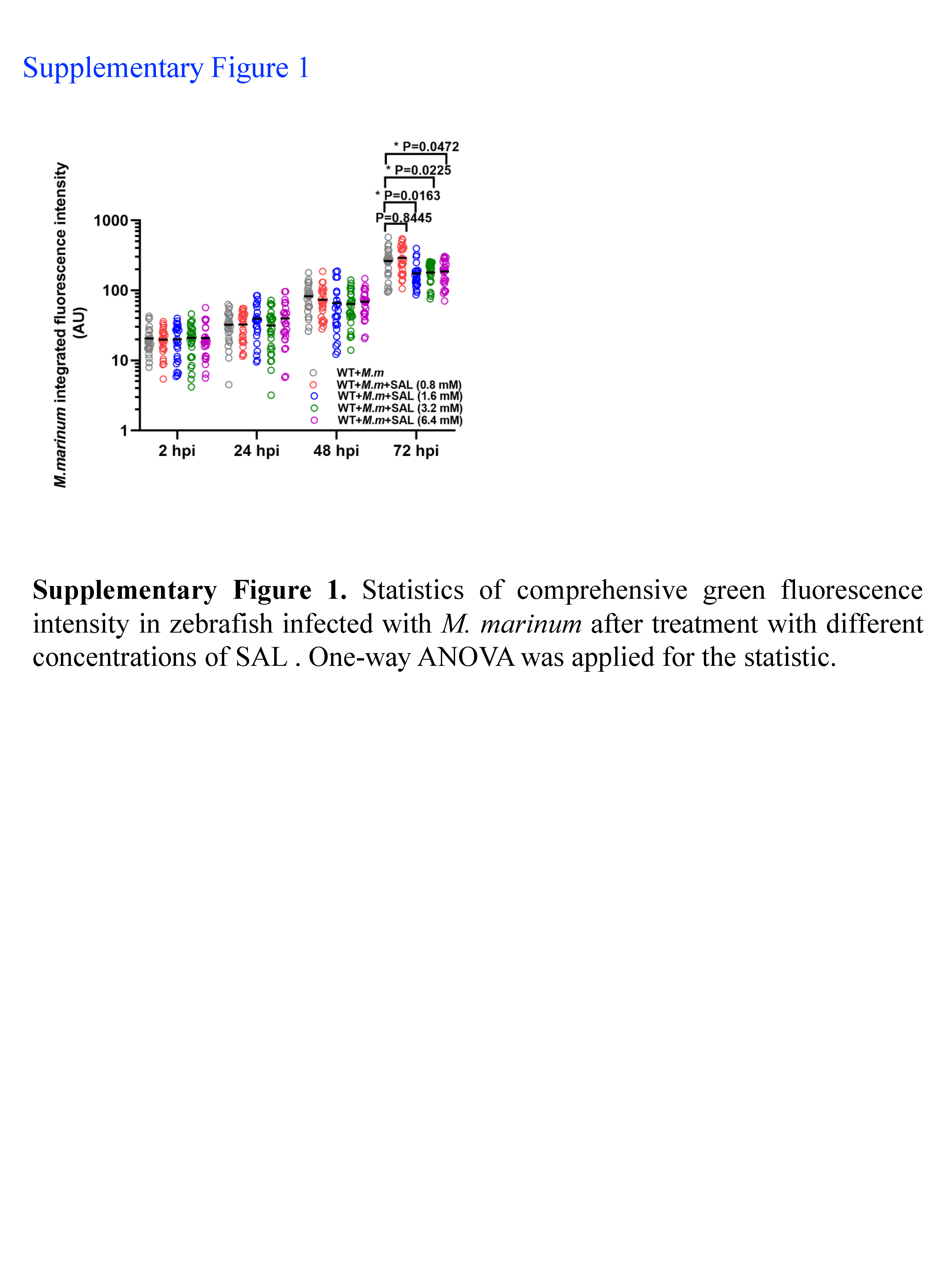

Supplement: Supplementary file 3 [file Image2.TIFF]
